# The Clinical Efficacy of Multidose Oritavancin: A Systematic Review

**DOI:** 10.3390/antibiotics12101498

**Published:** 2023-09-29

**Authors:** Giammarco Baiardi, Michela Cameran Caviglia, Fabio Piras, Fabio Sacco, Roberta Prinapori, Maria Luisa Cristina, Francesca Mattioli, Marina Sartini, Emanuele Pontali

**Affiliations:** 1 Pharmacology and Toxicology Unit, Department of Internal Medicine, University of Genoa, 16132 Genoa, Italy; giammarco.baiardi@edu.unige.it (G.B.); m.cameran.caviglia@galliera.it (M.C.C.); fabio.piras@galliera.it (F.P.); fabio.sacco@galliera.it (F.S.); 2 Clinical Pharmacology Unit, Ente Ospedaliero Ospedali Galliera, 16128 Genoa, Italy; 3 Department of Infectious Diseases, Ente Ospedaliero Ospedali Galliera, 16128 Genoa, Italy; roberta.prinapori@galliera.it (R.P.); emanuele.pontali@galliera.it (E.P.); 4 Operating Unit Hospital Hygiene, Ente Ospedaliero Ospedali Galliera, 16128 Genoa, Italy; maria.luisa.cristina@galliera.it (M.L.C.); marina.sartini@galliera.it (M.S.); 5 Department of Health Sciences, University of Genoa, 16132 Genoa, Italy

**Keywords:** oritavancin, pharmacokinetics, drug administration schedule, treatment outcome, multidose regimen, clinical pharmacology

## Abstract

Oritavancin (ORI) is a semisynthetic lipoglycopeptide approved as a single 1200 mg dose intravenous infusion for the treatment of acute bacterial skin and skin structure infections (ABSSSIs) caused by Gram-positive organisms in adults. The pharmacokinetic/pharmacodynamic (PK/PD) linear kinetic profile and long terminal half-life (~393 h) of ORI make it therapeutically attractive for the treatment of other Gram-positive infections for which prolonged therapy is needed. Multidose regimens are adopted in real-world clinical practice with promising results, but aggregated efficacy data are still lacking. A comprehensive search on PubMed/Medline, Scopus, Cochrane and Google Scholar databases was performed to include papers published up to the end of January 2023. All articles on ORI multiple doses usage, including case reports, with quantitative data and relevant clinical information were included. Two reviewers independently assessed papers against the inclusion/exclusion criteria and for methodological quality. Differences in opinion were adjudicated by a third party. From 1751 potentially relevant papers identified by this search, a total of 16 studies met the inclusion criteria and were processed further in the final data analysis. We extracted data concerning clinical response, bacteriologic response, mortality and adverse events (AEs). From the 16 included papers, 301 cases of treatment with multidose ORIs were identified. Multidose regimens comprised an initial ORI dose of 1200 mg followed by 1200 mg or 800 mg subsequent doses with a varying total number and frequency of reinfusions. The most often treated infections and isolates were osteomyelitis (148; 54.4%), ABSSSI (35; 12.9%) and cellulitis (14; 5.1%); and MRSA (121), MSSA (66), CoNS (17), *E. faecalis* (13) and *E. faecium* (12), respectively. Clinical cure and improvement by multidose ORI regimens were observed in 85% (231/272) and 8% (22/272) patients, respectively. Multidose ORI was safe and well tolerated; the most frequent AEs were infusion-related reactions and hypoglycemia. A multidose ORI regimen may be beneficial in treating other Gram-positive infections besides ABSSSIs, with a good safety profile. Further studies are warranted to ascertain the superiority of one multidose ORI scheme or posology over the other.

## 1. Introduction

Oritavancin (ORI) is a semisynthetic lipoglycopeptide antibiotic approved in 2014 by the US Food and Drug Administration (FDA) [1] and in 2015 by the European Medicines Agency (EMA) [2] as a single 1200 mg dose by intravenous infusion over 3 h for the treatment of acute bacterial skin and skin structure infections (ABSSSIs) caused by Gram-positive organisms in adults [1,2]. ORI is a derivative of the natural glycopeptide chloroeremomycin, which is similar to vancomycin [3].

ORI inhibits the transglycosylation step of cell wall biosynthesis in Gram-positive bacteria by binding to the D-alanyl-*D*-alanine (D-ala-D-ala) stem terminus of peptidoglycan precursor lipid II, a mechanism shared with all glycopeptides and lipoglycopeptides. ORI differs from chloroeremomycin by the addition of an *N*-alkyl-p-chlorophenylbenzyl substituent on the disaccharide, enhancing the activity against vancomycin-susceptible and vancomycin-resistant *Enterococci* (VSE and VRE) [3,4]. Moreover, it inhibits the transpeptidation step of cell wall synthesis by binding to the bridging segment of the cell wall peptidoglycan, a secondary binding site that has not been demonstrated for vancomycin [5,6,7]. These mechanisms of action result in bactericidal activity through loss of integrity, depolarization and permeabilization of the wall of Gram-positive bacteria, leading to rapid cell death [8,9].

ORI exhibits potent activity against Gram-positive organisms, including methicillin-susceptible (MSSA), methicillin-resistant (MRSA) and vancomycin-resistant (VRSA) *Staphylococcus aureus*, as well as VSE and VRE [10]. The current ORI minimum inhibitory concentration (MIC) susceptibility breakpoint (EUCAST) for *S. aureus* isolates is 0.125 mg/L, and it is 0.25 mg/L for Streptococcus groups A, B, C and G and for the *Streptococcus anginosus* group [11].

After intravenous infusion, ORI expresses extensive tissue distribution with an estimated total volume of distribution (Vd) of approximately 1 L/kg and high protein-binding properties (~85%) [12]. The greatest uptake is reported in the liver, followed by the kidney, spleen and lungs (59–64%, 2.7%, 1.8% and 1.7%, respectively) in healthy humans [13].

ORI is not metabolized, and its major route of elimination is the kidney; however, renal elimination appears to be a slow process, with ORI clearance (CL) of <0.5 mL/minute. In addition, <5% and 1% of the dose were recovered in urine and feces, respectively, at 7 days after a single dose, making it unlikely that dosage adjustments will be needed in patients with renal or hepatic dysfunction [2,12].

ORI displays a linear kinetic profile with multiexponential decline and a terminal half-life (T_1/2_) of 393 h (>10 day) [12] depending on variable tissue accumulation. Interestingly in macrophages ORI intracellular concentrations of 200-fold the extracellular ones are reached [14].

Due to the long plasma T_1/2_, the time during which the concentration of drug in the plasma exceeds the MIC (T > MIC) and the relationship between the area that is under the plasma free drug concentration–time curve (fAUC) and the MIC of the specific microorganism causing the infection (fAUC/MIC) seem to represent the pharmacokinetic/pharmacodynamic (PK/PD) parameters that best correlate with efficacy in vivo [15].

ORI could display concentration-dependent killing properties in vivo, so its bactericidal activity may also correlate to the maximum plasma concentration (Cmax) [16]. ORI Cmax and AUC from zero to time infinity (AUC_0-∞_) in patients with ABSSSIs who received a single 1200 mg dose are 138 μg/mL and 2800 μg∙h/mL, respectively [2].

The antibacterial effect from the single 1200 mg dose regimen of ORI was tested in an in vitro PK/PD model over 72 h against the MRSA isolates [17]. These results provided further justification for the assessment of the single 1200 mg dose for treatment of ABSSSIs, which was investigated during the pivotal clinical trials SOLO I and SOLO II: global phase 3 noninferiority studies, using 7–10 days of twice-daily vancomycin as a comparator [18,19]. The SOLO trials demonstrated the noninferiority to the comparator and tolerability of ORI 1200 mg single-dose regimen in the treatment of ABSSSIs in all the efficacy endpoints [18,19].

From the SOLO studies’ pooled data safety analysis [20], an ORI single 1200 mg dose is well tolerated with a safety profile similar to that of vancomycin. The incidences of adverse events (AEs), serious adverse events (SAEs) and discontinuations due to AEs were similar for ORI (55.3, 5.8 and 3.7%, respectively) and vancomycin (56.9, 5.9 and 4.2%, respectively). The long half-life of ORI compared with that of vancomycin did not result in a clinically meaningful delay in the onset or prolongation of AEs.

Despite glycopeptide-related AEs of interest including hypersensitivity, infusion site reactions/phlebitis, vestibular toxicity/ototoxicity, hematologic effects and nephrotoxicity, their incidence was lower in the ORI safety population and, significantly, there were no changes in urinalysis parameters suggestive of a nephrotoxic effect ORI [20].

From the population pharmacokinetic (PopPK) analysis of SOLO trials, no dose adjustment for single 1200 mg ORI is required for patients with mild to moderate renal function impairment, mild to moderate hepatic impairment or based on age, gender, race, weight or diabetes status [21].

Of note, a relationship was observed for increasing ORI CL in a linear way for patients weighing > 80 kg, and it was determined that those weighing > 110 kg would require dose adjustments to maintain fAUC/MIC similar to that of patients weighing less [13,22].

ORI’s long half-life and broad Gram-positive activity justify the growing interest in the treatment of other infections caused by Gram-positive bacteria. Furthermore, ORI Vd and penetration into invasive sites such as lung and bone are favorable for exploring its use in complicated infections [15]. PK/PD properties and the safety profile of ORI, then, make it therapeutically attractive to offer therapeutic options beyond its single dose use. ORI use in the treatment of complicated infections such as bacteremia, endocarditis, pneumonia, osteomyelitis and surgical site infections is currently under investigation, and several case reports or case series exist [23]. Nevertheless, the severity and high relapse rates of these latter infections may require more than a single dose of treatment. There is no agreement on the necessary number of ORI administrations, or about their frequency. Multidose regimens have been employed in real-world clinical settings, but aggregated efficacy data are still lacking, and limited (or no) information is available about the optimal timing and dose for the second or the following administrations.

We aimed to evaluate published multidose ORI clinical experiences in various types of Gram-positive infections.

## 2. Results

**Figure 1:**
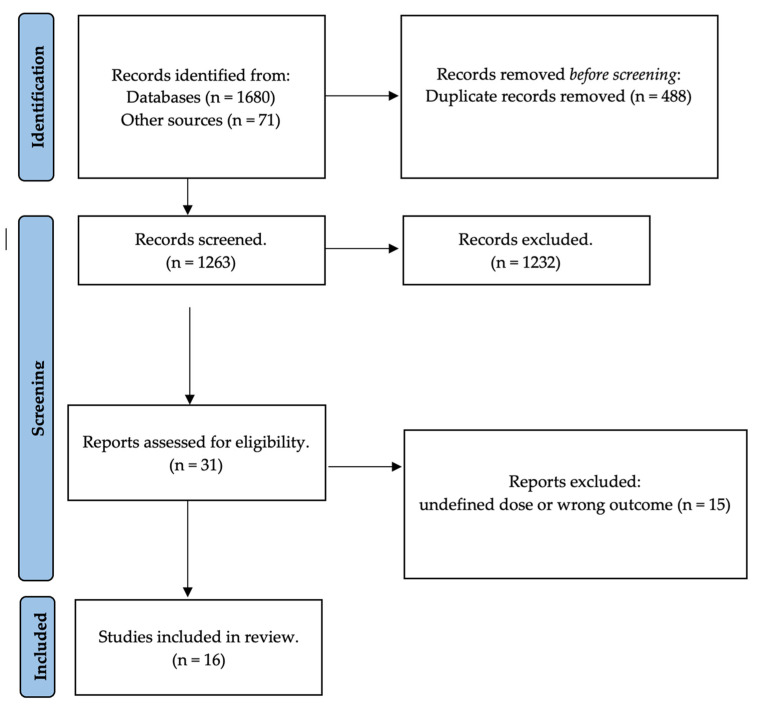
PRISMA 2020 diagram for study selection.

The initial query resulted in 1751 hits (specifically, 1680 articles from PubMed/MEDLINE and Scopus and 71 from other sources). After removal of duplicate items, the resulting list included 1263 non-redundant articles; then, 1232 were excluded because they did not meet the inclusion criteria for the study. Only 31 studies were retained in the qualitative synthesis, and 16 were finally evaluated for our systematic review (Figure 1).

According to our inclusion criteria, we selected 16 papers [24,25,26,27,28,29,30,31,32,33,34,35,36,37,38,39] for data extraction (see Supplementary Materials Table S1). They were all retrospective and most of them were case reports or case series (10/16; 62.5%). Table 1 summarizes identifiable pathogens that have been cultured and treated with ORI multidose regimens. Among treated pathogens, the most frequent species were *Staphylococci*, *Enterococci* and *Streptococci*, respectively. The most frequently treated infections were due to MRSA (121), followed by MSSA (66), coagulase-negative Staphylococci (CoNS) (17), *Enterococcus faecalis* (13) and *Enterococcus faecium* (12). The numbers of less frequently reported species are also given in Table 1.

The clinical outcomes of ORI multidose regimens are provided in Table 2 and Table 3 by etiology and site of infection, respectively. Direct comparison between the individual outcome, the isolated pathogen and the infection site was not possible, as this information was not reported by all the included studies, especially the largest ones. Overall, a clinical cure (C) was observed in 66.7% (58/87) and clinical improvement (I) in 26.4% (23/87) when a microbiologic isolate was available. *S. aureus* was the predominant pathogen with the highest success rate (81.5% MRSA, 75.0% MSSA), followed by CoNS (58.3%) and *Enterococci*.

The clinical success rate by site of infection was reported as C in 85.0% (231/272) and I in 8.0% (22/272) of treated patients. Among 272 patients with an identified site of infection, osteomyelitis (148) accounted for the majority of infections treated with ORI multidose regimens, with a cure rate of 89.2% (132/148), followed by ABSSSIs 82.9% (29/35), cellulitis 50% (7/14) and surgical wound infections 92.9% (13/14). Improvement rate by site of infection was 2.7% (4/148), 14.2% (5/35) and 50% (7/14) for osteomyelitis, ABSSSIs and cellulitis, respectively.

Failure (F) of multidose ORI was more common in wound infections (33.3%), bacteriemia (25%) and osteomyelitis (8.1%).

Multidose ORI regimens used in the evaluated studies are summarized in Table 4. In all cases, treatment started with a dose of 1200 mg followed by two possible subsequent multidose regimens based on 1200 mg or 800 mg (subsequent doses are always the same in each regimen). The number of repeated administrations and the frequency of subsequent infusions are reported in Table 4. Ten patients did not fit the classification reported in Table 4, because repeated administration of ORI occurred at arbitrary time points with arbitrary dose variations. Details on these latter regimens are reported in Table 5.

Safety and clinical outcomes of the different multidose schedules are also reported in Table 4. Multidose ORI was safe and well tolerated in most regimens. The most common AEs were infusion-related reactions (IRRs) and hypoglycemia.

A regimen which includes an initial ORI dose of 1200 mg with an 800 mg dose administration repeated between three and five times every ~7 days seems to be the multidose ORI regimen with the highest clinical success rate, independent of site of infection and isolated pathogen.

## 3. Discussion

ORI is one of three lipoglycopeptides for clinical use along with dalbavancin and telavancin [40].

The prolonged half-lives of ORI and dalbavancin opened the possibility for once weekly dosing. As such, there was great excitement for their potential role in avoiding or reducing hospitalization or length of stay for patients with ABSSSI. The current approved dose of ORI is a single 1200 mg intravenous infusion, for the treatment of ABSSSIs caused by Gram-positive organisms in adults. Its use in this setting has shown that ORI is not inferior to comparators, while showing advantages in reducing the rate of readmission and drug-related AEs [41].

A recent narrative review on the use of ORI in the treatment of non-ABSSSI infections has just been published, but the issue of multiple doses has not been discussed [23].

In this systematic review, we summarized the current evidence reported in the literature regarding the use of multidose ORI in the treatment of several infections, ABSSSIs and others.

Limited evidence has been reported in peer-reviewed papers so far on multidose ORI. Our work identified 16 papers on this use of ORI, and only about 300 subjects have been treated with this approach (Table S1). Unfortunately, available information on the included papers was not always complete. Thus, we reported, whenever possible, cultured pathogens (Table 1) and clinical outcomes of ORI multidose regimens by etiology and site of infection (Table 2 and Table 3). Some papers reported outcomes in relation to microbiological isolates and site of infection, while others, especially the ones with the largest cohorts, reported grouped information. This was the major limitation of our study, which prevented the possibility to specifically correlate etiology, site of infection and AEs with the dose of ORI and the number of repeated doses. In particular, the unavailability of unaggregated data from a cohort of 73 patients (see Table 4) treated with multidose ORI regimens, but without a clear distinction between the subsequent 1200 mg or 800 mg regimens, prevented our analysis by subtherapeutic groups from reaching statistical significance in order to recommend one multidose regimen over the other [37].

The spectrum of activity of ORI includes *Streptococcus* species, *E. faecalis*, *E. faecium* and *Staphylococcus* species (including *S. aureus*, both MSSA and MRSA), as well as *Clostridium* species. Due to the ability to bind to D-ala-D-lac (D-alanyl-*D*-lactate) stem terminus, ORI retains activity against VRE isolates (including vanA and vanB productor strains), heteroresistant vancomycin-intermediate *S. aureus* (hVISA), VISA and VRSA [42,43,44,45,46,47].Thus, as expected, this review identified studies where the most frequently treated pathogens were Staphylococci, Enterococci and Streptococci, including 121 MRSA, 2 VISA, 1 MRSE and 10 VR *E. faecium*. A clinical cure was observed in more than two thirds of cases (66.7%, 58/87); it exceeded 81% (22/27) for MRSA. When a favorable outcome, combining clinical cure and clinical improvement, was considered, it was well above 90% (i.e., 93.1%, 81/87), confirming ORI efficacy in infections due to Gram-positive bacteria. In particular, a favorable outcome was observed in 88.9% of MRSA cases (24/27), 100% of MSSA (16/16), 100% pf CoNS (12/12), 83.3% of VRE (5/6) and 100% of VISA (2/2); this is in line with what is reported in the literature [18,19,48,49,50,51].

In evaluating the selected peer-reviewed articles, a wide range of uses of multidose ORI was found, some of which, such as bacteremia and osteomyelitis, have been studied [23,34,36,52]. First, multidose ORI was employed in several cases of ABSSIs, cellulitis and other similar infections (69 cases). Nevertheless, multidose ORI was mainly used to treat osteomyelitis, septic arthritis and prosthetic joint infections (171 cases). Other interesting and challenging uses included surgical would infections (14), bacteremia (8), pneumonia (7) and endocarditis (7). A clinical cure was obtained in 85% of cases; it reached 100% for prosthetic joint infections, pneumonia and endocarditis. When a favorable outcome, combining clinical cure and clinical improvement, was considered, it was well above 90% (93%), confirming ORI efficacy in infections due to Gram-positive bacteria even in challenging sites of infection and clinical situations. These results are in line with what is highlighted by the review by Lupia et al. [23], who identified several advantages, many potential and yet unproved, in the use of ORI to treat endocarditis, bacteremia, prosthetic joint infections and osteomyelitis. In addition, it must be emphasized that our results show ORI efficacy in challenging clinical situations that is comparable to what is observed when ORI is used as a single shot in ABSSSIs [19,51,53].

This paper reports the largest collection of cases of Gram-positive infections treated with multidose ORI. The most surprising issue is that wide uncertainty exists on the dose and the number of repeated infusions administered during multidose regimens until cure, as well as on the frequency of infusion. We know that (i) the antibacterial activity of ORI seems to correlate better with overall drug exposure as measured by the fAUC via in vivo/in vitro PK models of infection, (ii) the key PK/PD parameter for clinical efficacy in humans is the fAUC/MIC ratio, and (iii) a single dose of ORI, which allows us to obtain high values of maximum plasma concentration (Cmax), results in greater antibacterial activity than fractionated doses. Indeed, ORI seems to have a concentration-dependent bactericidal action both in vitro and in vivo [15,16,54]. Therefore, a single initial dose, together with the PK characteristics of ORI (such as the long half-life), should allow for greater efficacy than fractionated doses in the clinical setting. On the contrary, there are few PK studies carried out on multidose ORI treatments [13,22]. Rose et al., using a PopPK model, report a high fAUC/MIC ratio for efficacy against Gram-positive organisms when ORI doses of 1200 and 800 mg are administered intravenously over 3 h and 1 week apart, compared with the single-dose regimen [55].

The safety profile of ORI has been previously established in the phase 3 SOLO trial [51]. Pruritus and diarrhea were most common and reported by 7% and 5% of the patients; fewer patients experienced treatment-emergent AEs that led to drug discontinuation (5.8%). Few patients experienced infusion-related reactions (3.8%). Since lipoglycopeptide and lipopeptide antimicrobial molecules interfere with some phospholipid-dependent coagulation markers, ORI has been shown to alter some coagulation tests, artificially modifying prothrombin time (PT), partial thromboplastin activated time (aPTT) and other tests for more than 120 h after infusion. The interference of ORI in these tests seems temporary, and the results revert to normal ranges within a few days after dosing [46,56].

Our results confirm the safety of ORI even when it is used as multidose ORI. The evaluated papers reported few or no AEs. When they were actually reported, they were mainly mild and the most frequent were back pain, IRRs, hypoglycemia, cytopenia and tachycardia (Table 4); their incidence was slightly different from what is reported in the literature [20].

This review shows that all authors approached a multidose ORI strategy with an initial dose of 1200 mg (as for the ABSSSIs: one-shot treatment), followed by either 1200 mg or 800 mg repeated doses (56 vs. 160 cases). A few authors did report exactly how many patients were treated with one subsequent dose or the other (1200 mg vs. 800 mg). The analysis of these two groups of dose regimens was further complicated by the fact that, independently of the site of infection, the number of following doses ranged from 1 to 32 and the frequency of dosing ranged from 7 to 28 days. Even if most cases received the following doses every approximately 7 days, the variability was extremely wide. Notwithstanding the excellent results obtained with all regimens and schedules, it was not possible to achieve clear comparisons among the several strategies employed in the different cases/cohorts to assess the superiority of one multidose schedule over the other.

All these considerations clearly call for urgent studies to better define PK/PD of multidose ORI regimens.

## 4. Materials and Methods

This systematic review of the literature was registered on the PROSPERO database (CRD42023458687) and reported following the Preferred Reporting Items for Systematic Reviews (PRISMA 2020) Statement [57] to ensure the current standards for systematic review reporting.

A comprehensive search on PubMed/Medline, Scopus, Cochrane and Google Scholar databases was performed. The following search terms were included: Oritavancin AND “off-label” OR “repeat* dose” OR “multiple dos*” OR “one week dose” from inception up to February 2023 using Medical Subject Headings (MeSH) terms as vocabulary, according to the National Center for Biotechnology Information (NCBI) nomenclature and guidelines and, where appropriate, a wild-card option.

Inclusion criteria were as follows: (1) research articles on *off-label* or use of multidose ORI with quantitative data and relevant information and (2) prospective or retrospective studies. Exclusion criteria were as follows: (1) articles not strictly related to the research query; (2) papers without sufficient information on dosage; and (3) research works not matching the PICOS criteria; all such articles were therefore excluded. No time filter or language filter was applied. For further details on the search strategy, see Table S2.

Six of the authors independently screened the literature. Any case of disagreement was solved by discussion until consensus was reached.

Articles were firstly selected based on title and abstract using the Rayyan platform for Systematic Review [58]. The full text of relevant research was then acquired and assessed. Each reference of the selected articles was checked in order to not miss any relevant articles. The risk of bias and the study quality were independently assessed by two researchers using the “The National Institutes of Health (NIH) quality assessment tool for case series studies”, “The National Institutes of Health (NIH) quality assessment tool for Observational cohort and cross-sectional studies” and “The National Institutes of Health (NIH) quality assessment tool for case–control studies”. All the included articles reported a “Good” quality rating. The authors then proceeded to independently read all the papers and implemented a database including the surname of the first author, the year and country of publication, the kind of study and the outcome. The clinical outcomes reported in this paper are extracted from the different cited papers. However, it was not always possible to provide a specific definition of clinical cure, either improvement or failure, since each evaluated paper had either a different definition or no definition.

## 5. Conclusions

This systematic review shows that multidose ORI could be a safe and effective strategy to treat several challenging infections due to Gram-positive pathogens, beyond ABSSSIs. However, great concerns remain about what the optimal long-term treatment scheme(s) is/are after the 1200 mg initial dose. At present, there appears to be no superiority of one multidose ORI scheme or dosage over the other in subsequent administrations (1200 mg vs. 800 mg), even with regard to dosing frequency. Further studies are needed to better compare the different multidose ORI schedules and posology in terms of clinical outcome and tolerability.

## 6. Registration and Protocol

This systematic review of the literature was registered on the PROSPERO database (CRD42023458687) and reported following the Preferred Reporting Items for Systematic Reviews (PRISMA 2020) Statement.

## Figures and Tables

**Table 1:** Etiological agents of infections treated with ORI.

Culture and Pathogen		*N*.
		
*Staphylococcus* spp.		
MRSA		121
MSSA		65
VISA		2
MRSE		1
CoNS		17
*Staphyloccoccus lugdunensis*		1
		
*Enterococcus* spp.		
*Enterococcus faecium*		12 (10 VRE)
*Enterococcus fecalis*		13
*Enterococcus gallinarum*		1
		
*Streptococcus agalactiae*		3
*Streptococcus pyogenes*		2
*Streptococcus anginosis*		2
*Streptococcus viridans*		2
*Streptococcus* group A/F		1
*Streptococcus* group G		1
*Streptococcus mitis*		1
		
*Corynebacterium* spp.		1
*striatum*		3
*not striatum*		1
		
*Skin Mixed flora*		3
		
*Peptostreptococcus* spp.		1
*Finegoldia magna*		1
		
*Bacillus* spp.		1
		
*Lactobacillus* spp.		1
		
*Propionibacterium acnes*		1
*Propionibacterium not acnes*		1
		
Gram +		1
		
NG or N/A		10
		

Abbreviations: MRSA = methicillin-resistant *Staphylococcus aureus*; MSSA = methicillin-susceptible *Staphylococcus aureus*; VISA = vancomycin-intermediate *Staphylococcus aureus*; MRSE = methicillin-resistant *Staphylococcus epidermidis*; CoNS = coagulase-negative *Staphylococci*; VRE = vancomycin-resistant *Enterococcus;* Gram + = Gram-positive bacteria not better defined; NG = no growth; N/A = culture results not available.

**Table 2:** Etiology and outcome.

Etiology	Outcome				
	Total	C% (N)	I% (N.)	F% (N.)	LTF% (N.)
MRSA	27	81.5% (22)	7.4% (2)	7.4% (2)	3.7% (1)
MSSA	16	75.0% (12)	25.0 (4)		
CoNS	12	58.3% (7)	41.7% (5)		
Not cultured/NG	10	50.0% (5)	40.0% (4)	10.0% (1)	
VRE	6	50.0% (3)	33.35 (2)	16.7% (1)	
*Enterococcus faecalis*	3	66.7% (2)	33.3% (1)		
*Streptococcus pyogenes*	2	50% (1)	50% (1)		
VISA	2	100% (2)			
*Skin Mixed flora*	2		50% (1)	50% (1)	
*Bacillus* sp.	1	100% (1)			
Gram +	1	100% (1)			
MRSE	1	100% (1)			
Streptococcus group A/F	1	100% (1)			
*Corynebacterium* sp.	1		100% (1)		
*Enterococcus faecium*	1		100% (1)		
*Staphyloccoccus lugdunensis*	1		100% (1)		
**Total Outcome**	**87**	**66.7% (58)**	**26.4% (23)**	**5.8% (5)**	**1.1% (1)**

Abbreviations: N: number; MRSA = methicillin-resistant *Staphylococcus aureus*; MSSA = methicillin-susceptible *Staphylococcus aureus*; CoNS = coagulase-negative Staphylococci; VRE = vancomycin-resistant *Enterococcus faecium;* VISA = vancomycin-intermediate Staphylococcus aureus; MRSE = methicillin-resistant *Staphylococcus epidermidis*; Gram + = Gram-positive bacteria not better defined; NG = no growth; N/A = culture results not available. C = clinically cured; I = improvement; F = failure; LTF = lost to follow-up.

**Table 3:** Outcomes by site(s) of infection.

Site(s) of Infection	Outcome				
	Total	C% (N.)	I% (N.)	F% (N.)	LTF% (N.)
Osteomyelitis	148	89.2% (132)	2.7% (4)	8.1% (12)	
ABSSSIs	35	82.9% (29)	14.2% (5)	2.9% (1)	
Cellulitis	14	50% (7)	50% (7)		
Surgical wound infection	14	92.9% (13)			7.1% (1)
Osteomyelitis and Septic arthritis	10	100% (10)			
Prosthetic joint infection	10	100% (10)			
Bacteremia	8	37.5% (3)	25% (2)	25% (2)	12.5% (1)
Pneumonia	7	100% (7)			
Endocarditis	7	100% (7)			
Other	5	80% (4)			20% (1)
Diabetic foot infection	3	100% (3)			
Septic arthritis	3	33.3% (1)	66.7% (2)		
Wound	3	66.7% (2)		33.3% (1)	
Infusion line infection	2	100% (2)			
Endovascular prosthesis	1		100% (1)		
Liver abscess	1		100% (1)		
Peritonitis	1	100% (1)			
**Total Outcome**	**272**	**85.0% (231)**	**8.0% (22)**	**5.9% (16)**	**1.1% (3)**

Abbreviations: N: number; ABSSSIs: Acute bacterial skin and skin structure infections; C = clinically cured; I = improvement; F = failure; LTF = lost to follow-up.

**Table 4:** Oritavancin treatment schemes, including safety and efficacy.

Initial Dose	Multidose Regimen	Number of Repeated Administrations	Frequency of Dosing	Adverse Events	Clinical Outcomes (n. of Treated Patients)
1200 mg	1200 mg				
		1	~7 days	None reported	C (7)
					I (3)
					F (1)
		3		None reported	C (2)
					I (1)
		5		None reported	C (4)
		6		None reported	C (3)
		8		Mild nausea	C (1)
		9		None reported	I (1)
			
		3	7–14 days	None reported	I (1)
		4			I (1)
		5			C (1)
			
		1	9–12 days	None reported	C (3)
				Infusion-related rigors and sharp back pain and spasms during infusion	I (2)
				None reported	F (1)
		3		None reported	C (2)
			
		1	14 days	None reported	C (11)
					I (3)
		2		None reported	C (1)
					F (1)
		4			I (1)
			
		2	14–21 days	Hearing loss	C (1)
				None reported	F (1)
			
		4	14–28 days	None reported	C (1)
			
		1	~28 days	None reported	C (2)
1200 mg	800 mg	1	~7 days	None reported	C (2)
					I (2)
		2		None reported	C (2)
				Substernal chest pain with shortness of breath	I (1)
		3		None reported	C (118)
				None reported	I (1)
				3 hypoglycemia and 2 tachycardia	F (16)
		4		None reported	C (11)
				Anemia, leukopenia (1)	I (1)
		5		None reported	C (2)
					I (1)
		7		None reported	I (1)
		11		None reported	I (1)
			
		1	14 days	Infusion-related reaction	I (1)
				
				
	1200 or 800 mg	1–32	~7 days	Back pain (3), rash (1), flushing (1), pruritus (1), headache (1), shortness of breath (1), pancytopenia (1)	C (34)
					I (34)
					F (5)

Abbreviations: C = clinically cured; I = improvement; F = failure.

**Table 5:** Unclassifiable ORI multidose regimens (single cases).

1200 mg on day 1, 52 and 90 (no AEs, C) 1200 mg on day 1 and 72 (no AEs, C) 1200 mg on day 1 and 34 (no AEs, C) 1200 mg on day 1, 14, 44 and 148 (no AEs, C) 1200 mg on day 1, 36, 73 and 147 (no AEs, C) 1200 mg on day 1, 14, 28, 70, 84 and 113 (no AEs, C) 1200 mg on day 1 and 9, then 800 mg cycled every week for 6 weeks (no AEs, C) 1200 mg on day 1, 3 and 5, then 1200 mg cycled every week for 6 weeks (no AEs, C) 1200 mg on day 1 and 7, then 800 mg cycled every week for 7 weeks (no AEs, C) 1200 mg on day 1, then 800 mg cycled every week for 11 weeks followed by 1200 mg after 11 days, then 800 mg cycled every week for 5 weeks (no AEs, I)

Abbreviations: no AEs = no reported adverse events; C = clinically cured; I = improvement.

## Data Availability

Not applicable.

## Supplementary Materials

The following supporting information can be downloaded at: https://www.mdpi.com/article/10.3390/antibiotics12101498/s1, Table S1: Included Articles; Table S2: PICOS search strategy adopted in the present systematic review.
