# Supplementary material for: The Clinical Efficacy of Multidose Oritavancin: A Systematic Review
Source: Antibiotics (Basel). 2023 Sep 29;12(10):1498. doi: 10.3390/antibiotics12101498 (PMC10604328; doi:10.3390/antibiotics12101498)
Supplement: Supplementary file 1 [file antibiotics-12-01498-s001.zip › antibiotics-2552331-supplementary.pdf]

**Table S1.** Included Articles

| First Author et al. (Year) | Country of Origin | Type of Study | N. of Patients with ORI multidose regimen on total ORI treatment |
|---|---|---|---|
| Stewart, Cassie L et al. 2017 [24] | USA | retrospective chart review | 10/10 |
| Delaportas, Dino J et al. 2017 [25] | USA | case report | 1/1 |
| Schulz, Lucas T et al. 2018 [26] | USA | retrospective cohort study | 17/44 |
| Foster, Rachel A et al.2018 [27] | USA | case report | 1/1 |
| Ruggero, Michael A et al. 2018 [28] | USA | case report | 1/1 |
| Antony, Suresh J et al. 2017 [29] | USA | case report | 2/2 |
| Nguyen, Jullian P et al. 2020 [30] | USA | case report | 1/1 |
| Ahiskali, Aileen et al. 2020 [31] | USA | case series | 11/24 |
| Chastain, Daniel B et al 2019[32] | USA | case series | 12/12 |
| Dahesh, Samira et al. 2019 [33] | USA | case report | 1/1 |
| Redell, Mark et al. 2019 [34] | USA | retrospective observational study | 32/440 |
| Morrisette, Taylor et al. 2019 [35] | USA | retrospective cohort study | 2/14 |
| Van Hise, Nicholas W et al. 2020 [36] | USA | retrospective observational study | 134/134 |
| Brownell, Lauren E et al. 2020 [37] | USA | retrospective observational study | 73/73 |
| Johnson, Jennifer A et al. 2015 [38] | USA | case report | 1/1 |
| Terrero Salcedo A D et al 2018 [39] | USA | case series | 2/5 |

**Table S2.** PICOS search strategy adopted in the present systematic review.

| Search Strategy | Details | |
|---|---|---|
| **Search string** | (Oritavancin [MeSH Terms]) AND (off-label) OR (repeat* dose) OR (multiple dos*) OR (one week dose) | |
| **Inclusion criteria** | P (patients/population): | outpatients and hospitalized patient |
| | I (intervention/exposure): | Subjects receiving multiple doses of oritovancin (off-label use) |
| | C (comparisons/comparators): | single dose |
| | O (outcome): | cure |
| | S (study design): | prospective or retrospective studies, editorial, commentaries, expert opinions, letters to editor, review articles |
| **Databases** | PubMed/MEDLINE, Scopus | |
| **Exclusion criteria** | Experimental studies investigating in vitro or animal model.<br>Study design: articles with insufficient details | |
| **Time filter** | None (from inception) | |
| **Language filter** | None (any language) | |
